# Impact of muscular symptoms and/or pain on disease characteristics, disability, and quality of life in adult patients with hypophosphatasia: A cross-sectional analysis from the Global HPP Registry

**DOI:** 10.3389/fendo.2023.1138599

**Published:** 2023-03-27

**Authors:** Kathryn M. Dahir, Priya S. Kishnani, Gabriel Ángel Martos-Moreno, Agnès Linglart, Anna Petryk, Cheryl Rockman-Greenberg, Samantha E. Martel, Keiichi Ozono, Wolfgang Högler, Lothar Seefried

**Affiliations:** ^1^ Division of Endocrinology and Metabolism, Vanderbilt University Medical Center, Nashville, TN, United States; ^2^ Division of Medical Genetics, Department of Pediatrics, Duke University Medical Center, Durham, NC, United States; ^3^ Departments of Pediatrics and Pediatric Endocrinology, Hospital Infantil Universitario Niño Jesús, Madrid, Spain; ^4^ Department of Pediatrics, Universidad Autónoma de Madrid, Madrid, Spain; ^5^ Centro de Investigación Biomédica en Red Fisiopatología Obesidad y Nutrición (CIBERobn), Instituto de Salud Carlos III, Madrid, Spain; ^6^ Department of Endocrinology and Childhood Diabetes, Paris Saclay University, AP-HP and INSERM, Paris, France; ^7^ Department of Global Medical Affairs, Alexion, AstraZeneca Rare Disease, Boston, MA, United States; ^8^ Department of Pediatrics & Child Health and Biochemistry & Medical Genetics, University of Manitoba, Winnipeg, MB, Canada; ^9^ Department of Epidemiology, Alexion, AstraZeneca Rare Disease, Boston, MA, United States; ^10^ Department of Pediatrics, Osaka University, Suita, Osaka, Japan; ^11^ Department of Paediatrics and Adolescent Medicine, Johannes Kepler University Linz, Linz, Austria; ^12^ Orthopedic Department, University of Würzburg, Würzburg, Germany

**Keywords:** 6MWT, rare diseases, alkaline phosphatase, SF-36, health-related quality of life, mobility, fracture, pseudofracture

## Abstract

**Introduction:**

Hypophosphatasia (HPP) manifests in adults as fractures/pseudofractures, pain, muscle weakness, and other functional impairments. Better phenotypic disease characterization is needed to help recognize disability and treat patients with HPP.

**Methods:**

Baseline/pretreatment demographic, clinical characteristic, and patient-reported disability/health-related quality-of-life (HRQoL) data from adults (≥18 y) in the Global HPP Registry (NCT02306720) were stratified by presence of overt skeletal manifestations (skeletal group) versus muscular/pain manifestations without skeletal manifestations (muscular/pain group) and summarized descriptively. Disability was measured using the Health Assessment Questionnaire–Disability Index (HAQ-DI), and HRQoL using the 36-item Short Form Health Survey (SF-36v2).

**Results:**

Of 468 adults, 300 were classified into the skeletal group and 73 into the muscular/pain group. The skeletal group had a higher median age at baseline (50.1 vs 44.4 y; *P*=0.047) but a lower median age at first HPP manifestation (12.3 vs 22.1 y; *P*=0.0473), with more signs and symptoms (median, 4 vs 3; *P*<0.0001) and involved body systems (median, 3 vs 2; *P*<0.0001) than the muscular/pain group. More patients in the skeletal group required any use of mobility aids (22.6% vs 3.5%, respectively; *P*=0.001). Six-Minute Walk test distances walked were similar between groups. SF-36v2 and HAQ-DI scores were similar between groups for physical component summary (n=238; mean [SD]: 40.2 [11.0] vs 43.6 [11.2]; *P*=0.056), mental component summary (n=238; mean [SD]: 43.6 [11.3] vs 43.8 [11.8]; *P*=0.902), and HAQ-DI (n=239; median [minimum, maximum]: 0.4 [0.0, 2.7] vs 0.3 [0.0, 2.1]; *P*=0.22).

**Conclusion:**

Adults with HPP experience similar QoL impairment regardless of skeletal involvement.

**Registration:**

https://clinicaltrials.gov/ct2/show/NCT02306720 and https://www.encepp.eu/encepp/viewResource.htm?id=47907, identifier NCT02306720; EUPAS13514.

## Highlights

Hypophosphatasia is a rare, inherited disorder that can affect the bones and teeth and can also cause muscular symptoms and/or pain in different patients. Data from many patients with hypophosphatasia have been entered into the Global HPP Registry. To learn more about the effects of bone symptoms and pain on quality of life, disability, and mobility in patients with HPP, registry data from 300 adults with bone symptoms were compared with data from 73 adults with muscular symptoms and/or pain but no bone symptoms. Results of this analysis suggest that hypophosphatasia caused considerable disability and lower quality of life, even in patients with no overt bone manifestations. In addition, patients with bone symptoms had experienced symptoms earlier in life than patients with muscular symptoms and/or pain and, therefore, had more severe symptoms possibly because of the longer time they had the disease. Some patients had experienced other complications of hypophosphatasia, including worsening in organ function and body systems, by the time their data were entered into the registry. These patients also more commonly relied on mobility aids, such as walkers, canes, crutches, wheelchairs, or scooters. Overall, patients with hypophosphatasia had more disability and lower quality of life than the general population. Because the complications of hypophosphatasia can change and progress over time, adults with this disease should meet regularly with their physicians and other health care providers to discuss the complications and how hypophosphatasia affects their lives.

## 1 Introduction

Hypophosphatasia (HPP; Online Mendelian Inheritance in Man [OMIM] code 146300) is a rare, inborn, inherited metabolic disease caused by deficient activity of the enzyme tissue-nonspecific alkaline phosphatase (TNSALP) (1). HPP is caused to a large degree by the buildup of the TNSALP substrate pyrophosphate and a decrease in production of pyridoxal from pyridoxal 5′-phosphate, an important component of neurotransmitter synthesis (2, 3). Because TNSALP plays a key role in bone mineralization, osteomalacia is considered a hallmark of HPP (2). Deficient mineralization in HPP can manifest clinically as insufficiency fractures or pseudofractures (4, 5). In both children and adults, TNSALP deficiency also can lead to broader musculoskeletal and systemic manifestations, the etiology of which is incompletely understood (4, 5). HPP disease presentation is varied and progressive, and our understanding of the disease continues to evolve. Whilst considered a bone disease, HPP is associated with other clinical features. In adults, HPP can manifest as muscle weakness, fatigue, pain, dental abnormalities, abnormal gait, chondrocalcinosis (pseudogout), arthropathy, and fractures/pseudofractures, among others (2, 5–7). Patients also may have a history of rickets, premature loss of deciduous teeth, craniosynostosis, seizures in infancy, or failure to thrive (7, 8).

Because HPP has a heterogeneous clinical presentation, misdiagnosis and delays in diagnosis often occur (7). In addition, adults with HPP can have a high burden of disease, often suffering from pain and, in some cases, requiring assistance with mobility (6). These factors contribute to decreased health-related quality of life (HRQoL) (6). An improved understanding of the presentation and impact of different HPP disease phenotypes may enable clinicians to better identify, treat, and support patients with HPP.

While skeletal manifestations and muscular/pain disease manifestations of HPP likely both contribute to poor function, disability, and reduced HRQoL in adult patients, the relevance of muscular pain/weakness in the absence of skeletal signs remains uncertain. Currently, only small case series and cross-sectional studies focused on the “bone phenotype” describe the impact of muscle pain/weakness among patients with HPP, and none discriminate between patients with and without overt skeletal signs (9–14). The aim of this study was to compare baseline/pretreatment data from patients in the Global HPP Registry (7) who had any skeletal manifestations with those from patients who had muscular/pain manifestations but no overt skeletal manifestations affecting their functional status and HRQoL.

## 2 Materials and methods

### 2.1 Data collection

The Global HPP Registry is an observational, prospective, multinational study that was initiated in 2014 (NCT02306720;EUPAS13514) (7). As part of routine clinical practice, physicians collected the following data from consenting adults (≥18 y) or their consenting parent or guardian at the time of enrollment into the registry: clinical course, signs, symptoms, and/or complications of HPP and burden of disease (7). Data were based on medical records available to the registry investigator and patient/parent recall (7). The Global HPP Registry collected HPP manifestations and assigned them to the appropriate body system category (ie, skeletal, muscular, dental, neurologic, renal, constitutional/metabolic, rheumatologic, respiratory, or pain) (6, 7).

Baseline medical history data of eligible patients were analyzed. Baseline refers to the time of registry enrollment for patients who were never treated with asfotase alfa (Strensiq^®^, Alexion Pharmaceuticals, Inc., Boston, MA, USA) or time of initiation of treatment with asfotase alfa.

### 2.2 Study population

Patients with a diagnosis of HPP confirmed by serum alkaline phosphatase (ALP) activity that was under the age- and sex-adjusted reference ranges and/or had an *ALPL* (HGNC ID 438) variant (including only pathogenic/likely pathogenic variants and variants of unknown significance) were eligible for participation in the Registry regardless of age, age of HPP onset, or prior or current asfotase alfa treatment status.

Because HPP affects primarily the musculoskeletal system, patients were grouped into 2 phenotypic categories based on the expert opinion of the Registry Advisory Committee: patients whose phenotype included specific skeletal manifestations regardless of muscular and/or pain manifestations (skeletal group) and those with muscular and/or pain manifestations without skeletal manifestations (muscular/pain group). Skeletal manifestations were defined as medical history of rickets, osteomalacia (if a bone biopsy was performed), recurrent or poorly healing fractures/pseudofractures, any fracture, chronic bone inflammation, bone marrow edema, calcific periarthritis, pseudogout, chondrocalcinosis, ectopic calcifications, abnormally shaped chest, abnormally shaped skull, bowing of long bones in legs or arms, scoliosis, and/or club foot deformity. Muscular/pain manifestations were defined as the absence of any of the skeletal manifestations described above and with a medical history of chronic muscle or generalized body pain, weakness, abnormal gait, fatigue, fibromyalgia, bone pain severe enough to limit activities, and/or bone pain severe enough to require pain medication. Patients who had skeletal manifestations in addition to muscular issues/pain were assigned to the skeletal group. Patients who did not have skeletal or muscular/pain manifestations were excluded from this analysis.

### 2.3 Assessments

Additional data captured for adults included measures of disability (assessed by the Health Assessment Questionnaire–Disability Index [HAQ-DI]) and HRQoL (assessed by the 36-item Short Form Health Survey version 2 [SF-36v2]). The SF-36v2 (QualityMetric, Inc, Lincoln, RI, USA) is a generic 36-item questionnaire that measures HRQoL *via* scores in 8 domains: vitality, physical functioning, bodily pain, general health perceptions, physical role functioning, social role functioning, emotional role functioning, and mental health (15, 16). Each domain has 2 to 10 questions, with a maximum total score of 100; lower scores indicate worse health status. The HAQ-DI consists of 24 questions and measures physical ability in 8 categories: dressing, arising, eating, walking, hygiene, reach, grip, and activities. HAQ-DI scores range from 0 to 3, with 0 representing function without any difficulty and 3 representing inability to do the task. For each of the 8 categories, the overall category score is equal to the highest item score in that category. Summed category scores are averaged to determine the final HAQ-DI score, and the maximum total score is 3.

Mobility was assessed by quantifying the use of mobility aids as well as through the 6-Minute Walk Test (6MWT). The 6MWT assesses ambulatory capacity by measuring the distance a person can walk on a hard, flat surface over a period of 6 minutes (17). The 6MWT has been validated as a reliable indicator of physical function in patients with HPP (18). For patients who did not perform the 6MWT, the reason was not collected (eg, nonambulatory). The use of mobility aids was evaluated by measuring the percentage of patients in each group that required any, part-time, or periodic use of mobility aids such as walkers, canes, crutches, wheelchairs, or scooters.

### 2.4 Statistical analyses

Data are summarized using descriptive statistics. The n values with available data on respective measures may be different than the study population N values based on sign and symptom manifestations. Continuous variables are reported as median (minimum [min], maximum [max]) and mean (standard deviation [SD]). Prevalence ratios and 95% confidence interval (CI) were calculated for HPP signs and symptoms by body system for patients with any skeletal symptoms compared with patients with only muscular and/or pain symptoms. Age of onset, number of signs and symptoms per patient, need for aid devices, and number of body systems affected per patient were compared between groups using the nonparametric Wilcoxon rank-sum test.

## 3 Results

### 3.1 Patients

Of 468 adults in the Global HPP Registry with a confirmed HPP diagnosis, 300 had a phenotype that included skeletal manifestations (skeletal group), and 73 had muscular/pain without skeletal manifestations (muscular/pain group). A total of 95 patients were excluded: 37 had signs and symptoms that were not skeletal or muscular/pain in nature, and 58 had signs and symptoms that were skeletal or muscular/pain in nature but did not occur prior to baseline. Demographics and baseline characteristics are shown in **Table 1**. Patients in the skeletal group had a trend toward a higher median age at baseline (*P*=0.160) but a lower median age at earliest HPP manifestation (*P*=0.0473) than those in the muscular/pain group. At baseline, the median number of signs and symptoms per patient was higher in the skeletal group than in the muscular/pain group (*P*<0.0001). Similarly, patients in the skeletal group also had more body systems affected per patient at baseline (*P*<0.0001). The number of patients with dental signs and symptoms at baseline was higher in the skeletal group than in the muscular/pain group, particularly early loss of primary teeth (prevalence ratio: 2.02; 95% CI: 1.45–2.60, *P*=0.0087). Most (n=236, 78.7%) patients in the skeletal group had muscle or pain symptoms.

**Table 1:** Demographics and baseline characteristics of study participants.

Characteristic	Skeletal Group (n = 300)	Muscular/Pain Without Skeletal Group (n = 73)	*P* Value^a^
Sex			0.374
n	300	73	
Female, n (%)	221 (73.7)	50 (68.5)	
Male, n (%)	79 (26.3)	23 (31.5)	
Age at baseline (y)			0.160
n	300	73	
Median (min, max)	50.1 (18.3, 81.2)	44.4 (19.3, 72.8)	
Age at earliest manifestation of HPP (y)			0.0473
n	208	44	
Median (min, max)	12.34 (−0.02, 75.3)	22.12 (0.0, 72.8)	
Age at HPP diagnosis (y)			
n	256	61	0.4837
Median (min, max)	43.21 (−0.03, 78.88)	40.09 (1.51, 72.81)	
Number of signs and symptoms per patient			<0.0001
n	282	73	
Median (min, max)	4 (1, 20)	3 (1, 7)	
Dental signs and symptoms, n (%)			
n	300	73	
Any	178 (59.3)	35 (47.9)	0.0779
Early loss of primary teeth^b^	91 (30.3)	11 (15.1)	0.0087
Other	157 (52.3)	32 (43.8)	0.1928
HPP onset, n (%)			0.0186
n	299	72	
Perinatal/infantile onset	10 (3.3)	2 (2.8)	
Juvenile onset	126 (42.1)	16 (22.2)	
Pediatric onset, specific type unknown	29 (9.7)	6 (8.3)	
Adult-onset HPP	95 (31.8)	34 (47.2)	
Unknown	39 (13.0)	14 (19.4)	
Number of body systems impacted per patient			<0.0001
n	282	73	
Median (min, max)	3 (1, 8)	2 (1, 5)	

a *P* values were calculated by Wilcoxon rank-sum test to test the statistical difference between groups. Prevalence ratios (95% CI) for the dental signs and symptoms suggest that a history of Any dental signs and symptoms (PR=1.24 [0.99, 1.50]), and in particular Early loss of primary teeth [PR=2.02 (1.45, 2.60)] are more common in the skeletal group vs muscular/pain without skeletal group. Other dental PR=1.20 (0.92, 1.48).

b Excludes patients <6 months at age of enrollment.

CI, confidence interval; HPP, hypophosphatasia; max, maximum; min, minimum; PR, prevalence ratio.

### 3.2 HRQoL assessment

**Figure 1:**
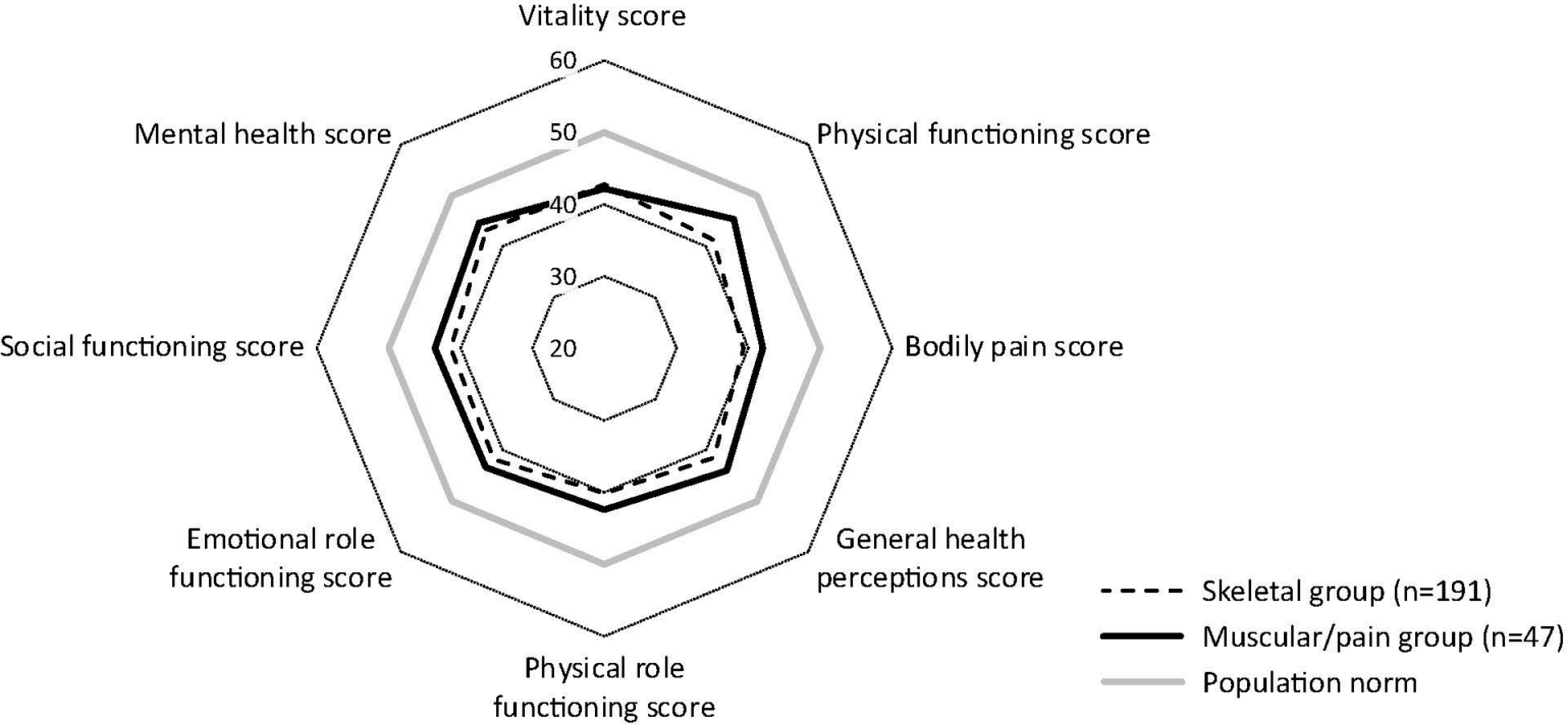
SF-36v2 domain scores at baseline. Mean scores are shown. The SF-36v2 scale is 0 to 100; 50 is average for the general population. SF-36v2, 36-item Short Form Survey version 2. Mean scores are shown; scale 0–100 (50 is average, and lower scores represent more disability. Mean (SD) scores for each domain were as follows for skeletal vs muscular/pain groups, respectively: vitality, 42.8 (10.7) vs 42.2 (11.5), *P*=0.720; physical functioning, 41.3 (10.9) vs 45.4 (10.7), *P*=0.022; bodily pain, 39.3 (10.3) vs 42.0 (11.7), *P*=0.112; general health perceptions, 41.4 (10.8) vs 44.0 (12.0), *P*=0.141; physical role functioning, 40.1 (11.6) vs 42.4 (11.8), *P*=0.233; emotional role functioning, 41.8 (12.3) vs 43.4 (12.6), *P*=0.409; social role functioning, 41.4 (12.1) vs 43.6 (13.3), *P*=0.282; and mental health, 43.3 (10.5) vs 44.6 (10.8), *P*=0.470.

HRQoL assessment, performed using the SF-36v2, was available for 238 patients (skeletal group, n=191; muscle/pain group, n=47). SF-36v2 summary scores are shown in **Table 2**. **Figure 1** shows SF-36v2 domain scores. Compared with the general population, which scores 50 for each of the 8 domains, patients in both study groups displayed lower HRQoL in every domain. SF-36v2 physical component and mental component summary scores did not differ between the skeletal and muscular/pain groups. The individual SF-36v2 domain scores, except the physical functioning score, did not differ between the skeletal and muscular/pain groups.

**Table 2:** SF-36v2 summary scores at baseline.

Characteristics	Skeletal Group (n = 300)	Muscular/Pain Without Skeletal Group (n = 73)	*P* Value
SF-36v2 results available, n	191	47	
SF-36v2 domain scores, mean (SD)			
Physical component	40.2 (11.0)	43.6 (11.2)	0.056
Mental component	43.6 (11.3)	43.8 (11.8)	0.902
Vitality	42.8 (10.7)	42.2 (11.5)	0.720
Physical functioning	41.3 (10.9)	45.4 (10.7)	0.022
Bodily pain	39.3 (10.3)	42.0 (11.7)	0.112
General health perceptions	41.4 (10.8)	44.0 (12.0)	0.141
Physical role functioning	40.1 (11.6)	42.4 (11.8)	0.233
Emotional role functioning	41.8 (12.3)	43.4 (12.6)	0.409
Social role functioning	41.4 (12.1)	43.6 (13.3)	0.282
Mental health	43.3 (10.5)	44.6 (10.8)	0.470

SD, standard deviation; SF-36v2, 36-item Short Form Survey version 2.

### 3.3 Disability assessment

**Figure 2:**
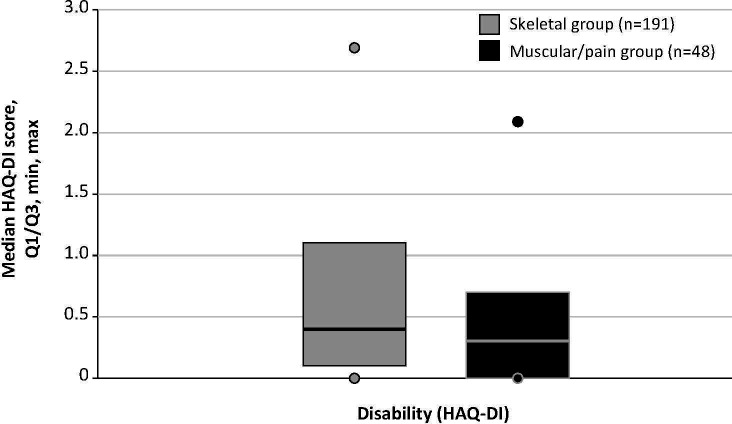
HAQ-DI scores among patients with and without skeletal manifestations. HAQ-DI score range is 0 (without any difficulty) to 3 (unable to do). HAQ-DI, Health Assessment Questionnaire–Disability Index. Lines indicate median scores; boxes, quartiles (Q) 1 and 3; dots, minimum and maximum.

Disability assessment data captured from the HAQ-DI questionnaire were available for 239 patients (skeletal group, n=191; muscular/pain group, n=47). Details are shown in **Figure 2**. The median (min, max) HAQ-DI score for the skeletal group was 0.4 (0.0, 2.7), and the score for the muscular/pain group was 0.3 (0.0, 2.1; *P*=0.22).

### 3.4 Mobility and fatiguability assessments

**Figure 3:**
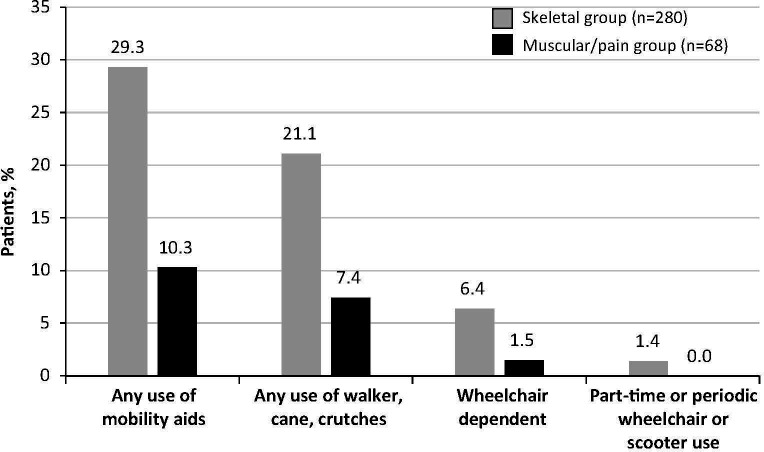
Use of assistive devices or mobility aids at baseline. Percentages of patients in the skeletal and muscular/pain groups with any or some use of mobility aids are shown.

A comparative analysis of the use of assistive devices or mobility aids among skeletal and muscular/pain groups is shown in **Figure 3**. A significantly larger proportion of patients in the skeletal group required any use of mobility aids (29.3% vs 10.3%, respectively; *P*=0.001). Specifically, a larger proportion of patients in the skeletal group had used a walker, a cane, or crutches (21.1% vs 7.4%; *P*=0.009), and part-time or periodic use of a wheelchair or scooter was infrequent and exclusive to the skeletal group (1.4%). A minority of patients were wheelchair dependent at baseline in both groups (6.4% vs 1.5%; *P*=0.14).

**Figure 4:**
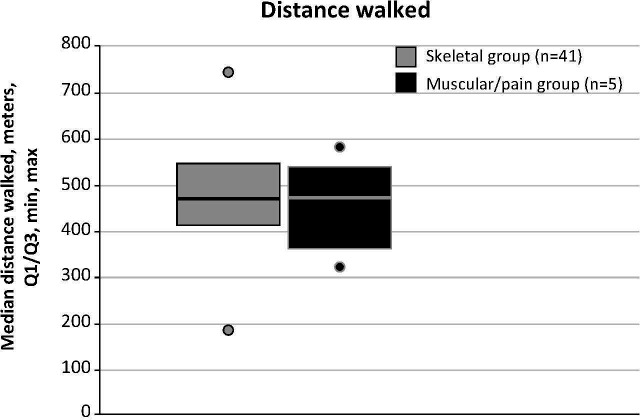
6-Minute Walk Test distance walked at baseline. In healthy adults, the 6-Minute Walk Test distance typically ranges from 400 to 700 m. Lines indicate median scores; boxes, quartiles (Q) 1 and 3; dots, minimum and maximum distance walked.

Results from the 6MWT are shown in **Figure 4**. Median distances walked on the 6MWT were comparable between the skeletal and muscular/pain groups, although the number of patients in the muscular/pain group was small. Patients in the skeletal group (n=41) walked a median (min, max) of 465 (180, 740) m, while those in the muscular/pain group (n=5) walked 466 (316, 580) m (*P*=0.855). Overall, of patients who completed the 6MWT, 9 out of 41 (22%) in the skeletal group and 2 out of 5 (40%) patients in the muscular/pain group walked less than 400 m.

## 4 Discussion

This analysis of adult patients in the Global HPP Registry suggests that patients with HPP can experience considerable disability and compromised QoL regardless of whether they have any overt skeletal manifestations of the disease. Because this analysis was cross-sectional, it is conceivable that, given the evolving and progressive nature of HPP, the muscular/pain group also may manifest overt skeletal signs over time (2, 5). Compared with patients in the muscular/pain group, those in the skeletal group had earlier symptom onset, more manifestations and body systems involved at baseline, and more frequently used mobility aids. Although the trend toward a greater median age at baseline in the skeletal group in this analysis may explain part of this difference, it is also possible that these patients with skeletal involvement included a larger number of those with particularly severe and early disease manifestations before skeletal maturity.

Both the skeletal group and muscular/pain group had similar 6MWT performances, disability factors other than physical functioning, and QoL measures. This comparable degree of disability and general QoL highlights the importance of including muscular issues and pain in the diagnostic workup of patients with suspected HPP. This approach has the potential to prevent a missed diagnosis or a misdiagnosis in patients with relevant disease burden devoid of overt bone manifestations and to promote better disease recognition in the clinical setting.

Patients with HPP have been reported to have poor HRQoL resulting from heavy disease burden, including pain and disease manifestation in multiple body systems (19). This poor QoL is also seen in our data regardless of the presence of skeletal disease manifestations; patients in both study groups scored worse than people in the general population in every domain of the SF-36v2. Supporting the findings of our study, Weber and colleagues also reported that patients with HPP had scores lower than those of the general population on a modified version of the SF-36v2 (9). Together, these data further indicate the detrimental effects of HPP on patient QoL and underscore the importance of investigating the impact of deficient ALP activity on other tissues and metabolic processes not immediately related to bone and mineralization.

In the present study, the degrees of disability, as indicated by HAQ-DI scores, were similar between patients with and without skeletal HPP manifestations. These data suggest that the presence of skeletal manifestations is not the main or only determinant of poor function, disability, and reduced HRQoL in adults with HPP and that muscular/pain manifestations also play a role.

The 6MWT is a reliable and valid measurement of functional capacity in children, adolescents, and adults with HPP (18). In patients with HPP who completed the 6MWT, distances were similar between the skeletal group and the muscular/pain group, and median distances were within the lower range of the typical 400 to 700 m walked by healthy individuals (20, 21). Nine of 41 patients (22%) in the skeletal group and 2 out of 5 (40%) patients in the muscular/pain group walked less than 400 m. Mobility aids, however, were more prevalent among patients with skeletal manifestations, suggesting that this patient population experiences impaired movement to a greater extent.

There was a preponderance of female patients in the study population assessed, so the proportion of female participants was slightly larger than in previous reports on other adult HPP cohorts. Considering the autosomal inheritance and largely balanced gender distribution in pediatric HPP cohorts, there is no reasonable pathophysiologic explanation for this preponderance. Potential reasons for this finding may include an enhanced health awareness and readiness among female patients to seek professional evaluation for nonspecific symptoms such as those frequently observed in adults with HPP. The larger proportion of females may also represent ascertainment bias because the data may reflect diagnosis in osteoporosis clinics; females undergo screening for osteoporosis and other medical check-ups more regularly than males, during which a low ALP level may be a coincidental finding. Another factor that may have contributed to the exceptionally larger proportion of females in this study, even when compared with retrospective adult HPP cohorts, may be an increased preparedness of females to participate in health-related scientific projects.

There were a few limitations to this study. Because the Global HPP Registry is purely observational, with data collected from medical records during standard medical practice, there may be variations in the availability of data due to differences in standard of care from site to site and for different patients based on severity of disease. To mitigate issues due to data variation, all attempts were made to obtain missing data (including patient data at baseline) in the Global HPP Registry and to verify the accuracy of key parameters. Small numbers in some patient groups, particularly those in the muscular/pain group with the 6MWT data available, also may limit our ability to draw conclusions from these data. Registry data do not explicitly indicate whether patients were unable to complete the 6MWT, so data were available only for those who completed the assessment. In addition, the history of clinical manifestations, while obtained from medical records, may be influenced by recall bias if events were reported to the clinician by the patient. Long recall intervals may have reduced the accuracy of the data, particularly for those with less severe manifestations. Assessments used in this analysis focused on general disability and QoL, may not accurately capture the burden of disease for patients with predominantly dental manifestations, and may be affected by assessment not including all patients being assessed. Additionally, muscle or pain symptoms in the patients in the skeletal group could have impacted HRQoL and disability scores.

## 5 Conclusion

The results of this analysis show that adults with HPP, regardless of age of onset or overt skeletal involvement, experience serious disease burden. Patients with skeletal involvement appear to have a greater number of body systems affected than those with muscular/pain manifestations but no skeletal manifestations. Overall, these data add to the emerging understanding of HPP manifestation in adults, indicating that assessment of adults with HPP should regularly include assessment of muscular symptoms and pain in addition to skeletal evaluation.

## Data availability statement

The original contributions presented in the study are included in the article/supplementary material, further inquiries can be directed to the corresponding authors.

## Ethics statement

This study involved human participants and was reviewed and approved by the institutional review board (or local equivalent) of participating study sites and is being conducted in accordance with International Conference on Harmonisation Good Clinical Practice Guidelines and the Declaration of Helsinki. All patients and/or their parent/legal guardians provided written informed consent and approval to release medical records before participation. The patients/participants provided their written informed consent to participate in this study.

## Author contributions

Conceptualization and methodology: KD, LS, PK, AP, WH, AL, GM-M, KO, SM, and CR-G. Data curation: KD, LS, PK, WH, AL, GM-M, KO, and CR-G. Formal analysis: SM. Writing – original draft, review, and editing: KD, LS, PK, AP, WH, AL, GM-M, KO, SM, and CR-G. All authors contributed to the article and approved the submitted version.
